# Newly Discovered Rustrela Virus: Current State of Knowledge About the Etiological Agent of Feline “Staggering Disease”

**DOI:** 10.3390/pathogens14090851

**Published:** 2025-08-27

**Authors:** Anna Słońska, Ilona Stefańska, Ewelina Kwiecień, Dorota Chrobak-Chmiel

**Affiliations:** Department of Preclinical Sciences, Institute of Veterinary Medicine, Warsaw University of Life Sciences, Ciszewskiego 8, 02-786 Warsaw, Poland; ilona_stefanska@sggw.edu.pl (I.S.); ewelina_kwiecien1@sggw.edu.pl (E.K.); dorota_chrobak@sggw.edu.pl (D.C.-C.)

**Keywords:** rustrela virus, feline staggering disease, nonsuppurative meningoencephalomyelitis, zoonotic potential

## Abstract

The rustrela virus (RusV), a recently discovered member of the *Matonaviridae* family and a close relative of the rubella virus, has emerged as the etiological agent of “staggering disease”—a progressive neurological disorder primarily affecting domestic cats and other mammals. Characterized by nonsuppurative meningoencephalomyelitis, RusV infection manifests with clinical signs such as ataxia, seizures, and behavioral abnormalities. First identified in 2020, RusV has since been detected in various mammalian species across Europe and, more recently, in North America. This review provides a comprehensive summary of the current knowledge of RusV, including its taxonomy, genomic structure, host range, transmission hypotheses, clinical and histopathological features, and diagnostic challenges. Although the potential for zoonotic spillover has not yet been confirmed, it highlights the need for increased surveillance and further research. As an emerging neurotropic virus with potential for cross-species transmission, RusV may represent a significant concern for veterinary medicine and public health.

## 1. Introduction

“Staggering disease” (SD) is a neurological disease affecting domestic cats (*Felis catus*), first identified in Sweden in the 1970s [1,2,3]. The name, derived from the Swedish term “vingelsjuka,” highlights the disease’s most prominent clinical symptom: ataxia of the hind limbs [4]. Additional cases of the disease were reported in the 1990s near Vienna, Austria. Despite affecting domestic cats for nearly fifty years, the causative agent has remained unidentified for a long time [5,6]. Initial studies suggested that the disease was caused by the Borna disease virus (BDV), but subsequent research failed to confirm this hypothesis [3,4,7]. Only recently, the rustrela virus (RusV), a close relative of the rubella virus (RuV), which causes human measles, has been identified as the cause of the nonsuppurative encephalomyelitis associated with the “staggering disease” [8]. Multiple recent and retrospective studies have confirmed the presence of RusV in the brain tissue of various mammalian zoo species, including a donkey (*Equus asinus*), a capybara (*Hydrochoerus hydrochaeris*), a red-necked wallaby (*Macropus rufogriseus*), and lions (*Panthera leo*) in Germany, all of which exhibited neurological disorders associated with severe encephalitis [8,9,10,11]. Detection of RusV RNA in wild yellow-necked field mice (*Apodemus flavicollis*) without apparent clinical signs and lack of inflammation suggests that these rodents may be considered as putative reservoir hosts of RusV [12].

This review thoroughly examines the rustrela virus (RusV), including its current taxonomy classification, genomic structure, host spectrum, clinical manifestation, histopathological features, and diagnostic tools. It also discusses the potential threat of zoonotic spillover, the current understanding of its epidemiology, and its broader implications for animal and human health. This review emphasizes the importance of continued surveillance and research into this emerging pathogen.

## 2. Etiology of “Staggering Disease”

A neurological disorder in cats, commonly referred to as “staggering disease” (SD), has been reported for nearly five decades and is characterized by nonsuppurative meningoencephalomyelitis. Clinically, the significant signs of the disease include gait disturbances such as hind leg ataxia and an inability to retract the claws, behavioral alterations, and, in some cases, hyperesthesia and seizures [1,2,6]. Although the etiological agent of the disease remained unidentified for decades, clinical and morphological features led to the conclusion that its origin was viral [13]. The Borna disease virus (BoDV), a member of the Mammalian 1 *Orthobornavirus* species (family *Bornaviridae*), was initially considered a potential causative agent due to its association with neurological disorders in various mammals, including humans [14]. Several studies have reported BoDV infection in domestic cats with neurological signs consistent with staggering disease, supporting its involvement as a possible etiological agent [7,15,16,17,18]. A study by Wensman et al., 2012, found that 89% of cats exhibiting neurological symptoms consistent with SD tested positive for BoDV-specific markers, including antibodies and/or viral RNA [17]. Although BoDV antigens or antibodies were frequently detected in clinical cases, earlier studies struggled to establish a clear and consistent correlation between BoDV detection and the manifestation of the disease.

A major breakthrough occurred with the application of metagenomic analyses, which identified RusV as being associated with feline staggering disease. The virus was detected in archival samples from 27 to 29 cats diagnosed with nonsuppurative meningoencephalomyelitis, confirming a strong association between RusV and SD. The presence of RusV was confirmed using a combination of molecular and immunohistological techniques, including reverse transcription quantitative polymerase chain reaction (RT-qPCR), in situ hybridization (ISH), and immunohistochemistry (IHC), all of which demonstrated both viral RNA and antigens in the infected tissue. Furthermore, RusV was frequently detected in wood mice (*Apodemus sylvaticus*), indicating their potential role as reservoir hosts. This finding aligns with the epidemiological pattern of SD, which is consistently associated with forested environments [2].

## 3. The Origin of Rustrela Virus

### 3.1. Discovery of Novel Rubiviruses

The first description of a new member of the *Matonaviridae* family was published in 2020, identifying the rustrela virus (RusV, Rubivirus strelense). The virus was named for its rubivirus-like genome and the German Strela Sound of the Baltic Sea, where it was initially discovered [8,19]. RusV was isolated from the brain tissue of various mammals at a zoo in northern Germany, including a donkey, a capybara, and a red-necked wallaby. All these animals developed severe, acute neurological disorders such as ataxia, convulsions, and lethargy before their deaths. Further investigations revealed the presence of RusV RNA in wild rodents, particularly in yellow-necked field mice (*Apodemus flavicollis*) found within the zoo and up to 10 km away. Although these rodents showed no symptoms or histological evidence of encephalitis, subsequent testing revealed RusV RNA in the brain tissue of yellow-necked field mice, suggesting a reservoir role and a potential spillover from mice to other susceptible species [8].

In addition to RusV, another member of the *Matonaviridae* family was discovered in cyclops leaf-nosed bats (*Hipposideros cyclops*) in Uganda. The ruhugu virus (RuhV, Rubivirus ruteetense) was identified in oral swabs from asymptomatic bats but has not been linked to any disease [8]. Similar to RusV, the virus’s name reflects its rubivirus-like genome, its host species (insectivorous bat, obuhuguhugu), and the geographical region (Ruteete Subcounty, Uganda) where it was first detected [19].

### 3.2. General Taxonomy of the Matonaviridae Family

In 2019, the International Committee on the Taxonomy of Viruses (ICTV) established a new family called *Matonaviridae*, reclassifying the rubella virus (RuV, Rubivirus rubella), which was previously a member of the family *Togaviridae* [19]. Rubella, formerly known as German measles, is an acute and highly contagious infection caused by RuV. It is characterized by symptoms such as fever, rash, adenopathy, and conjunctivitis. While most RuV infections are mild and self-limiting, infections during pregnancy can result in miscarriage or severe congenital disabilities [20]. Humans are the only known host of RuV, and no zoonotic transmission has been described.

Until 2020, RuV was considered the sole member of the genus Rubivirus since two relatives were recently identified in various mammals: rustrela virus (Rubivirus strelense), which infects wild rodents and zoo animals in Europe, and ruhugu virus (Rubivirus ruteetense), which infects bats in Africa (Table 1) [8,19]. Phylogenetic analysis has revealed that RuhV is the closest relative to RuV, sharing a highly conserved genomic architecture and protein sequences, which suggests similar, potentially conserved, membrane fusion capabilities and host entry mechanisms. In contrast, RusV forms a more distant lineage; however, within the genus Rubivirus, it retains key genomic features common to RuV and RuhV [8]. The discovery of RusV and RuhV has reshaped the understanding of rubivirus evolution, highlighting the presence of previously unrecognized rubella-like viruses in animal hosts.

Recent metagenomic and metatranscriptomic studies have identified matonavirus-like viruses in various ectothermic vertebrates, although these viruses remain unclassified by the ICTV. Notably, a rubivirus-related sequence was identified in the Guangdong Chinese water snake as part of a broad virological survey of vertebrates in China [21]. Similarly, the tiger flathead Matonavirus was detected in Australian marine fish through metatranscriptomic analysis [22], and a related virus, tetronarce Matonavirus, was identified from the transcriptome of the Pacific electric ray [23]. These findings underscore the expanding diversity of matonavirus-like agents in fish and reptile species.

**Table 1:** Taxonomy of the *Matonaviridae* family [19,24].

Realm	Kingdom	Phylum	Class	Order	Family	Genus	Species	Virus Name	Abbrev.
*Riboviria*	*Orthornavirae*	*Kitrinoviricota*	*Alsuviricetes*	*Hepelivirales*	*Matonaviridae*	*Rubivirus*	*Rubivirus* *rubellae*	Rubella virus	RuV
							*Rubivirus* *strelense*	Rustrela virus	RusV
							*Rubivirus* *ruteetense*	Ruhugu virus	RuhV

### 3.3. Host Range and Geographical Distribution of Rustrela Virus

RusV was identified in zoo animals, domestic cats, and wild rodents across Europe, with confirmed cases exclusively in Sweden, Germany, and Austria [2]. In Germany, a diverse range of mammalian hosts for RusV was documented, including equids, mustelids, rodents, and marsupials, indicating a broad spillover potential. Between July 2018 and October 2019, RusV RNA was detected for the first time in the brain tissue of a donkey, a capybara, and a Bennett’s tree-kangaroo (*Dendrolagus bennettianus*) housed in a zoological garden in northern Germany. In the case of donkey, viral RNA was also detected in the liver, suggesting the possibility of limited systemic spread [8]. However, no RusV antigen has been reported in extraneural tissues of affected animals to date, supporting the hypothesis that RusV is a strictly neurotropic virus [3]. All infected animals exhibited acute neurological symptoms prior to death, including ataxia, seizures, and lethargy [8].

Subsequent studies of wild rodents from the zoo and its surroundings confirmed the presence of viral RNA in the central nervous system (CNS) of yellow-necked mice, strengthening the hypothesis that these rodents can act as potential reservoir hosts. Interestingly, most infected mice did not exhibit symptoms of encephalitis and had low concentrations of RusV RNA in peripheral organs [8,12]. Further analysis of samples collected from different regions in Germany confirmed RusV RNA exclusively in samples collected from yellow-necked field mice (12.6%). No RusV RNA was detected in samples collected from other tested wild small rodents, including striped field mice (*Apodemus agrarius*, *n* = 94), wood mice (*n* = 55), house mice (*Mus musculus*, *n* = 57), Eurasian harvest mice (*Micromys minutus*, *n* = 22), Norway rats (*Rattus norvegicus*, *n* = 16), common voles (*Microtus arvalis*, *n* = 253), field voles (*Microtus agrestis*, *n* = 130), bank voles (*Myodes glareolus*, *n* = 207), common shrews (*Sorex araneus*, *n* = 73), Eurasian pygmy shrews (*Sorex minutus*, *n* = 5), Eurasian water shrews (*Neomys fodiens*, *n* = 2), and single samples collected from bicolored white-toothed shrew (*Crocidura leucodon*), lesser white-toothed shrew (*Crocidura suaveolens*), European hedgehog (*Erinaceus europaeus*), and European mole (*Talpa europaea*). Furthermore, additional cases of fatal RusV-associated meningoencephalitis diagnosed in 2022 also originated from this region. Viral RNA was detected in brain samples collected from a South American coati (*Nasua nasua*) and three red-necked wallabies (*Notamacropus rufogriseus*) from the zoo, as well as from local wildlife, including a wild Eurasian otter (*Lutra lutra*) and three yellow-necked field mice that were asymptomatic for encephalitis [10,11]. A retrospective study conducted in 2022 revealed the presence of RusV antigen and RNA in formalin-fixed, paraffin-embedded (FFPE) brain tissue from three lions that had died in the 1980s, housed in zoos in eastern and western Germany. These lions had been diagnosed with nonsuppurative meningoencephalitis at the time of their deaths.

Recent reports have identified the RusV in a new location in North America, with a confirmed case in a wild mountain lion (*Puma concolor*) in Colorado, USA. The affected mountain lion showed clinical symptoms, including severe hind leg ataxia and paresis, as well as histopathological lesions consistent with “staggering disease”, similar to cases previously reported in Europe. Phylogenetic analyses of the Colorado RusV strain revealed significant genetic divergence from European strains, raising questions about the virus’s evolutionary history and potential undiscovered reservoirs in North America [25,26].

In 2023, the association between RusV and “staggering disease” in cats was confirmed for the first time [2,4]. These studies examined fresh-frozen and FFPE brain and spinal cord samples from cats exhibiting clinical signs such as ataxia, hind leg weakness, and seizures, or histological findings of non-purulent encephalitis of unknown origin, indicative of “staggering disease.” The samples were sourced from Sweden, Austria, and Germany, spanning 1991–1993 (Austria) and 2017–2022 (Germany and Sweden) [2], as well as 1994–2016, predominantly from cats in Vienna and Lower Austria, with additional cases from other Austrian regions [4].

### 3.4. Potential Reservoirs and Transmission Pathways of Rustrela Virus

The natural reservoir of RusV remains uncertain, though current research suggests a significant role for rodents in its transmission, as they carry viral RNA without apparent symptoms of encephalitis. In northern Germany, yellow-necked field mice are considered potential reservoir hosts. However, in Sweden, RusV has only been detected in wood mice despite the co-existence of yellow-necked field mice in the same area. This discrepancy may reflect differences in species composition within the analyzed samples or indicate that RusV variants have adapted to alternative rodent hosts [2,12].

**Figure 1:**
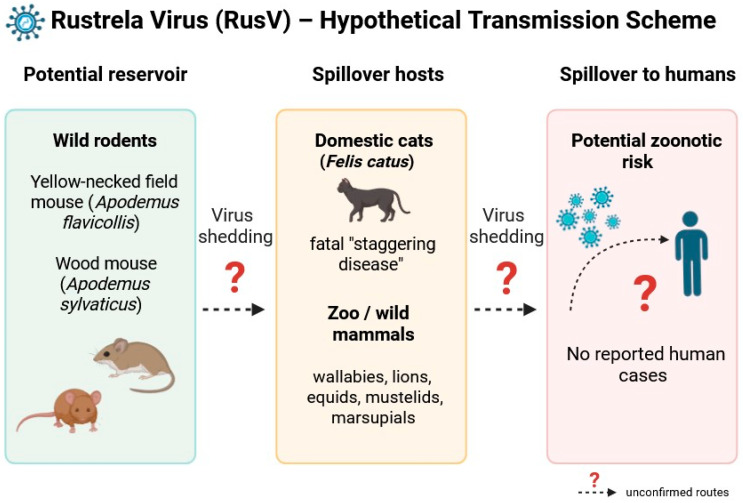
Schematic representation of the hypothetical transmission pathways of rustrela virus (RusV). Wild rodents—particularly the yellow-necked field mouse (*Apodemus flavicollis*) and the wood mouse (*Apodemus sylvaticus*)—are proposed as potential reservoir hosts. The exact mechanisms of virus shedding and interspecies transmission remain undetermined. Spillover infections have been documented in domestic cats (*Felis catus*) and various zoo or wild animals, including donkeys (*Equus asinus*), capybaras (*Hydrochoerus hydrochaeris*), red-necked wallabies (*Macropus rufogriseus*), lions (*Panthera leo*), and otters (*Lutra lutra*), leading to fatal nonsuppurative meningoencephalomyelitis. Although no human infections have been reported to date, the virus’s neurotropism, broad host range, and phylogenetic relationship to the rubella virus raise concerns regarding its zoonotic potential.

The route of RusV transmission is currently unknown, and viral shedding has not been conclusively documented. In zoo animals and yellow-necked field mice in Germany, RusV tissue tropism appears to be primarily confined to the CNS, with occasional detection of viral RNA in peripheral nerve fibers [8,10]. In RusV-positive yellow-necked field mice that do not have meningoencephalitis, RusV RNA was also found in the medullary cells of the adrenal gland, which is a modified sympathetic ganglion of the autonomic nervous system innervated by preganglionic sympathetic neurons originating in the spinal cord. This raises the question of whether the adrenal gland infection results from CNS involvement or occurs independently [12]. Bennet et al. also detected RusV in oral swabs and feces collected from yellow-necked field mice, suggesting the potential for transmission via oral secretions and excreta [8]. Currently, there is no evidence of direct transmission of RusV; instead, it appears to be transmitted from a reservoir host [4] (Figure 1). However, comprehensive data on the tissue distribution of RusV are still lacking and require further investigation.

### 3.5. Clinical and Histopathology Features of Rustrela Virus Infection

RusV-infected animals exhibited nonsuppurative meningoencephalomyelitis and a wide range of neurological symptoms affecting the locomotor system, consistent with the clinical profile of “staggering disease.” The clinical findings have been extensively documented in zoo animals, domestic cats, and wild felids, all presenting with similar clinical manifestations. The hallmark clinical sign of RusV infection is hind leg ataxia, typically accompanied by increased muscle tone, leading to distinctive movement disorders and staggering gait [2,4,8,9,10,11,25]. Additional neurological symptoms include an inability to retract claws, observed predominantly in infected domestic cats [2,4], as well as prolapse of the tongue, a symptom reported in RusV-infected lions [9]. Other commonly noted signs involve hyperesthesia [2,4], tremors, seizures, or convulsions, which may occur intermittently or progress to more severe, generalized neurological dysfunction [2,8,10]. Spastic paresis and paralysis are frequently observed in advanced cases, further contributing to the deterioration of motor control [11,25].

Beyond motor dysfunction, RusV infection is also associated with significant behavioral changes, which vary between individuals and species. Behavioral alterations range from increased vocalization and apathy or depression [11,12] to heightened affection [10] and, in rare cases, aggression [2]. The disease typically progresses over several days to weeks but can persist for over a year, often leading to euthanasia due to worsening conditions and welfare concerns.

Histopathological examination of RusV-infected animals revealed nonsuppurative meningoencephalitis, characterized by lymphohistiocytic perivascular cuffing, angiocentric immune cell infiltration, microglial nodules, and neuronal degeneration [10]. The inflammation predominantly affected the gray matter of the brainstem, cerebral cortices, hippocampus, and spinal cord, with less pronounced involvement of the cerebellum [2]. Neuronal degeneration in RusV-infected tissues was often accompanied by apoptotic cell death, as indicated by caspase-3 immunolabeling, particularly in the hippocampus and cerebral cortex [10]. IHC consistently detected CD3-positive T cells and IBA1-positive microglial cells, confirming an immune-mediated response [11]. Despite these inflammatory changes, viral RNA and antigens were often detected beyond areas of inflammation, suggesting that viral persistence and immune-mediated pathology contribute to disease progression [2].

In feline staggering disease, histological examination revealed perivascular lymphocytic and/or lymphohistiocytic cell infiltrates in the cerebral cortex and spinal cord, with a distribution pattern primarily in the brainstem, hippocampus, and neocortex [2,4]. The severity of inflammation varied among cases, ranging from mild to severe encephalitis, depending on the extent and density of the perivascular infiltrates [4]. Additionally, no Borna disease virus (BoDV) antigen was detected via immunohistochemistry, effectively ruling out BoDV as a potential cause of encephalitis [2,4]. This finding aligns with previous reports, which described inflammatory lesions in the CNS of experimentally BoDV-1-infected cats as predominantly affecting the white matter, in contrast to the gray matter-predominant pathology characteristic of ‘staggering disease’ caused by RusV [15].

Similar histopathological findings were observed in infected lions, red-necked wallabies, coatis, and otters, confirming RusV as the causative agent of encephalitis and further confirming RusV as the causative agent of encephalitis across a broad range of mammalian species [9,11].

### 3.6. Diagnostic Techniques and Challenges for Rustrela Virus Infection

Most reports on RusV are derived from retrospective analyses of brain and/or spinal cord samples that have been formalin-fixed and paraffin-embedded (FFPE). These samples were taken from animals diagnosed with multifocal lymphohistiocytic meningoencephalomyelitis or meningoencephalitis, accompanied by clinical signs consistent with the description of staggering disease. These specimens, collected between 1980 and 2022, were contributed by various laboratories in Sweden, Austria, and Germany [2,3,4,8,9,10,11,25,27]. Due to its relatively recent identification, diagnostic methods rely on molecular and histopathological techniques.

The identification of RusV RNA primarily relied on molecular techniques, with RT-qPCR and in situ hybridization (ISH) being the most widely used methods. The RT-PCR assay was designed to detect RusV RNA by targeting a highly conserved region at the 5′ terminus of the p150 gene, which encodes a non-structural polyprotein essential for viral replication [8]. As a component of the larger p200 polyprotein, p150 plays a crucial role in viral RNA synthesis, making it a highly reliable target for molecular diagnostics. Researchers, including Matiasek et al. and Bennett et al., carried out multiple sequence alignments of RusV genomes to identify the most conserved regions within p150, allowing for the optimization of primers and probe selection [2,8]. This process followed a bioinformatics-driven approach, with RusV sequences generated using the complete genome sequences available in GenBank, specifically those derived from the donkey (GenBank MN552442) and the cat (GenBank ON641041) [2,8].

In situ hybridization (ISH) has been employed to detect RusV RNA within infected tissues, enabling the visualization of viral distribution within specific regions of the CNS. One of the most commonly used ISH approaches for RusV detection is RNAscope, a highly sensitive assay that targets viral RNA with specific probes [27]. RNAscope has been successfully applied to formalin-fixed paraffin-embedded (FFPE) brain tissues from infected animals, revealing intense RusV RNA signals in neurons, astrocytes, Purkinje cells, and axons, which correspond to histopathological findings of nonsuppurative meningoencephalomyelitis [2,8,10,25]. In studies of feline staggering disease, ISH confirmed the presence of RusV RNA in 27 out of 29 affected cats, while no viral RNA was detected in healthy control animals [4]. Similarly, ISH has been instrumental in identifying RusV infection in other species, such as lions and wallabies [9,11].

Further confirmation of RusV infections was achieved through immunohistochemistry (IHC), which was effectively applied to brain samples from infected animals. Matiasek et al., 2023, described the development and use of the RusV capsid-specific monoclonal antibody 2H11B1 and demonstrated its value for the immunohistochemical detection of RusV antigen in infected tissues [2,9]. For monoclonal antibody production, a synthetic DNA fragment encoding a portion of the RusV capsid protein (amino acids 128–308) was expressed in human Expi293 cells, allowing the generation of hybridoma cells capable of producing RusV-specific monoclonal antibodies. The final antibody, 2H11B1, was validated through indirect ELISA and immunofluorescence assays on transfected RK-13 rabbit kidney cells, confirming its specificity for the viral capsid protein. Using IHC with 2H11B1, researchers successfully identified viral antigens in neurons, astrocytes, Purkinje cells, and axons, correlating with histopathological findings of nonsuppurative meningoencephalomyelitis. However, their studies also revealed that in cases where RT-qPCR yielded negative results from FFPE samples, IHC successfully confirmed the presence of RusV antigen. This suggests potential limitations in RNA preservation, primarily related to sample quality and viral load. One significant challenge is the loss of sensitivity in PCR assays and other molecular techniques due to using formalin-fixed paraffin-embedded (FFPE) tissues, representing most available samples [2]. The fixation and embedding process often leads to RNA degradation, reducing RT-qPCR efficiency and ISH and potentially resulting in false-negative results [9,28]. Additionally, the age of the samples may further compromise RNA integrity, making it difficult to detect low viral loads [4].

Currently, there are no serological tests that specifically identify RusV infection. The study by Bennett et al., 2020, found that the amino acid sequences of RuV, RuhV, and RusV are moderately to highly conserved within four putative B-cell epitopes, particularly in the E1 envelope glycoprotein, which plays a key role in host/cell fusion and immune recognition [8]. This similarity suggests that existing serological assays for anti-rubella antibodies may detect antibodies against RusV, RuhV, or other rubella-like viruses. However, their sensitivity and specificity remain unverified [8]. Future research should focus on standardizing serological methods that distinguish between RuV, RusV, and RuhV infections in different hosts, including the development of RusV-specific ELISA and virus neutralization tests (VNT), which would improve understanding of the virus’s epidemiology and potential zoonotic risk.

## 4. Genomic Structure and Sequence Characteristics of Rustrela Virus

The Matonaviridae family consists of small, enveloped viruses with positive-sense (+ss), non-segmented RNA genomes ranging from 9.6 to 10 kb [2]. Due to the lack of available virus isolates for RusV and RuhV, the structural and functional characterization of matonaviruses is primarily based on studies of the rubella virus (RuV) [12]. Moreover, sequencing analysis of RusV from organ samples remains challenging, resulting in a predominance of partial coding sequences in available datasets. As of July 2025, only 35 complete genome sequences have been deposited in GenBank (Supplementary Table S1). These include 20 sequences from yellow-necked field mice, five from domestic cats, three from wood mice, two from red-necked wallabies, and one sequence each from a donkey, South American coati, capybara, Eurasian otter, and cougar.

**Figure 2:**
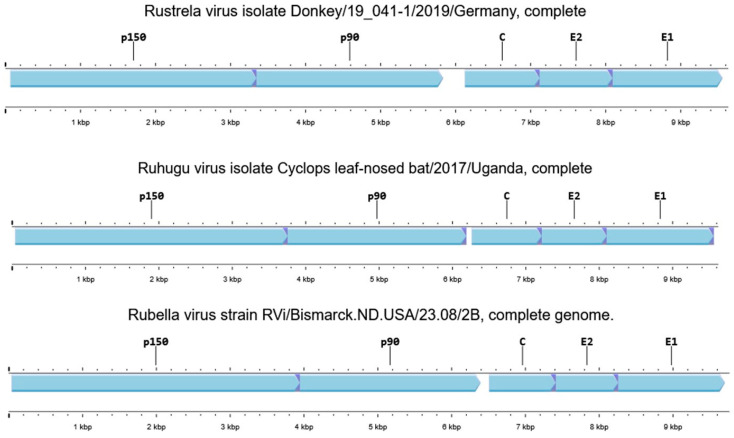
Comparative genome structure of rustrella virus (NC_076451.1), ruhugu virus (NC_076450.1), and rubella virus (NC_076948.1). The genome has two ORFs consisting of two non-structural polyproteins (nsPP), namely p90 and p150 proteins, and three structural proteins (sPP), including the C capsid protein and the two envelope glycoproteins E2 and E1 [29].

The genomic structures of RuV, RuhV, and RusV are highly conserved and feature two large open reading frames (ORFs), untranslated regions (UTRs) at the 5′ and 3′ termini, and an intergenic region (IR) separating the ORFs [8] (Figure 2). Among these, RusV has a genome length of 9631 nucleotides (strain donkey/19_041-1/2019/Germany), distinguished by having the longest intergenic region of 290 nucleotides and the most extended capsid-encoding sequence (332 codons) within the genus Rubivirus, setting it apart from its relatives [10]. The ORF located at the 5′ end (68-5833 nucleotide position) encodes a non-structural polyprotein (p200/nsPP), while the second ORF situated in the 3′ region (6124-9555 nucleotide position) encodes a structural polyprotein (p110/sPP) [10,19]. Both proteins, nsPP and sPP, are synthesized as polyprotein precursors of approximately 1921 and 1143 amino acid residues (aa), respectively, and are further processed into two or three polypeptides. The non-structural polyprotein (nsPP) is cleaved into two components: the protease p150 (150 kDa, 1093 aa), which is involved in replication complex formation, and the RNA-dependent RNA polymerase (RdRP) p90 (90 kDa, 828 aa), essential for viral genome replication. The sPP is processed into the capsid protein (C, 3321 aa), which plays a key role in genome packaging and virion assembly, and two envelope glycoproteins, E2 (324 aa) and E1 (487 aa), which are responsible for host receptor binding and membrane fusion during viral entry [10,12]. A membrane fusion protein, E1, is the main viral antigen.

The RusV genome exhibits a notably high guanine and cytosine (GC) content, averaging around 70 mol%, with localized peaks reaching 82 mol% [19]. Particularly elevated GC levels—up to 87 mol%—are observed within the intergenic region and the N-terminal portion of the capsid (C) protein coding sequence [30]. This has posed significant challenges to determining the complete RusV genome sequence. However, applying a target enrichment-based high-throughput sequencing (HTS) approach successfully enabled the generation of several high-quality genome sequences from presumed reservoir hosts and clinically affected spillover-infected animals [10].

The comparison of whole-genome sequences of 35 RusV strains indicated genome lengths ranging from 9564 to 9633 nucleotides in the analyzed strains. The highest variability occurs primarily in the intergenic region and the p150 gene, both of which are particularly rich in nucleotide deletions and insertions. The in silico analysis of five coding regions of RusV genomes of different strains showed the highest identity in nucleotide (≥76.1%) and amino acid (≥86.6%) sequences for the p90 coding region. The lowest identity (≥67.2% for nucleotide sequence, ≥67.9% for amino acid sequence) was noted for glycoprotein E2 (Table 2). Estimates of evolutionary divergence between RusV isolates are presented in Supplementary Table S2 (comparison of the whole-genome nucleotide sequences and coding regions for p150, p90, C, E2, and E1) and Supplementary Table S3 (amino acid sequence comparisons of these proteins).

Genomic divergence in RusV isolates can lead to significant functional implications, in particular affecting the ability to infect the host, replicate, transmit, and evade the host immune response. Viral E1 and E2 glycoproteins are exposed on the virus envelope and regulate the entry of RusV viruses into host cells [19]. E1 protein is crucial for membrane fusion, whereas E2 interacts with the cellular receptors and aids cell attachment [10,12,19]. Therefore, variability in glycoproteins can affect these processes and, in turn, influence viral infectivity and tissue or cell tropism. Furthermore, E1 is a major target of neutralizing antibodies, and variations in its structure can affect the effectiveness of the host’s immune response, potentially influencing the virus’s ability to evade immunity. Mutations in genes encoding non-structural proteins p150 and p90 can affect viral RNA synthesis, transcription, and overall replication efficiency. C protein is a multifunctional protein, and alterations in its structure or function due to mutation can lead to various changes, e.g., in virus replication and assembly inside the host cell. Moreover, genomic divergence can enable viruses to infect new host species or cell types, expanding the virus’s potential for transmission and disease, including the risk of spillover events. In essence, genomic divergence is a fundamental driver of viral evolution, impacting the virus’s ability to adapt to different host environments and evade immune response. However, there is a lack of research explaining how the genetic changes observed in various RusV strains influence their pathogenicity.

## 5. Phylogenetic Analysis

**Figure 3:**
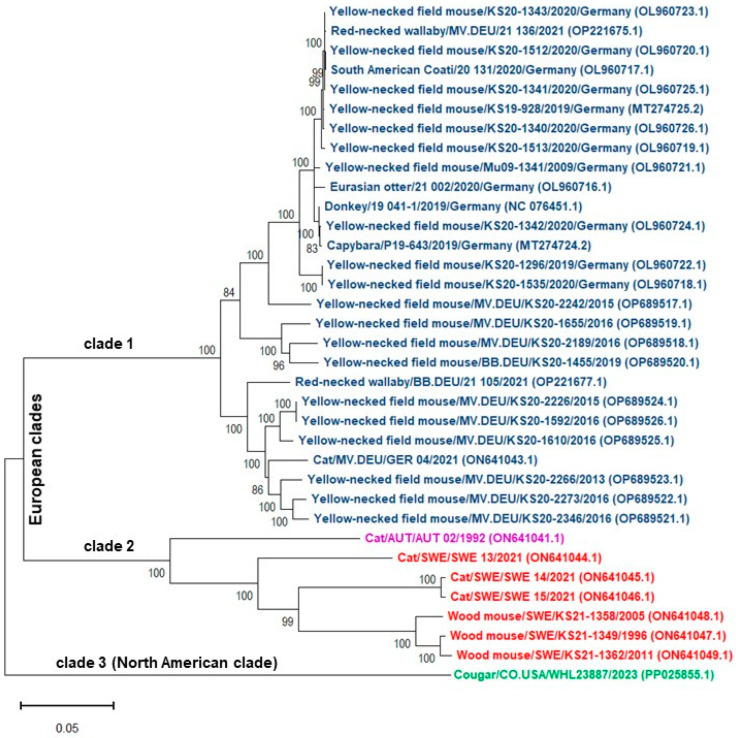
Phylogenetic analysis based on nucleotide alignment of the RusV whole genomes obtained from the NCBI GenBank database (accession numbers in parentheses). The names of virus strains were colored according to their country of origin, and included isolation host, sampling location, animal/strain ID, and sampling year. The analysis involved 35 genomes, and a total of 9710 positions were in the final dataset. All sequences were aligned using MUSCLE. Phylogenetic analysis was conducted in MEGA11 [31] using the Maximum Likelihood method and General Time Reversible model [32]. The scale bar indicates the number of substitutions per site. Bootstrap values calculated for 500 replications are shown next to the branches. GER—Germany, BB—Brandenburg, MV—Mecklenburg-Western Pomerania, AUT—Austria; SWE—Sweden, CO.USA—Colorado.

Phylogenetic analysis of whole-genome sequences revealed that all strains are grouped into three clearly distinct clades, each associated with a different geographical location (Figure 3). The first clade includes all RusV viruses originating from cats, yellow-necked field mice, and other animals from various regions in Germany, sharing at least 91.5% nucleotide sequence identity. The second clade comprises all RusV strains isolated from cats and wood mice in Sweden, with at least 85.8% nucleotide sequence identity. Additionally, the RusV strain from an Austrian cat clusters with these strains, showing ≥81.6% nucleotide identity. In contrast, the RusV strain isolated in the USA from a cougar is the most distantly related, exhibiting only 70.5–71.1% nucleotide sequence identity compared to the other strains. Cat isolates from Sweden showed 85.8% to 99.5% nucleotide sequence identity, whereas isolates from different countries shared between 76.5% and 82.2% identity. Greater than 92.4% and 96.7% nucleotide sequence identities were found among yellow-necked field mice and wood mice isolates, respectively.

## 6. Potential for Zoonotic Transmission of Rustrela Virus

The current prevalence of rustrela virus (RusV) in domestic cats and wildlife remains largely unknown. While RusV has been confirmed as the causative agent of staggering disease in cats, it has also been detected in other European species, including equids, mustelids, rodents, and marsupials, suggesting a notably broad host range. So far, documented cases of RusV infections associated with staggering disease have mainly occurred in isolated instances across Central and Northern Europe, particularly in northern Germany, Austria, and southern Sweden, suggesting a potentially limited geographic distribution [2,3,4,8,9,10,11] and a likely lack of research to detect the virus in other countries. However, most recent studies indicate that the virus also occurs in North America in the USA [25]. Whether RusV-associated meningoencephalomyelitis occurs more widely across Europe or affects other animal species remains unknown and warrants further investigation.

It is essential to highlight that RusV displays a broad host range despite its seemingly limited occurrence. This contrasts with its close relative, the rubella virus (RuV), which is highly contagious and globally distributed but restricted to humans as its sole host [20]. Although no human infections have been reported, the virus’s demonstrated ability to cross species barriers and cause severe neurological disease in a diverse range of mammals underlines the importance of continued surveillance.

Additionally, the presence of highly conserved regions in the envelope protein shared between RusV and RuV suggests the potential for RusV to infect humans [8,20]. While RusV exhibits some amino acid similarity with RuV in these proteins, it also displays notable differences that may influence viral function and host range. Although direct experimental data on the roles of RusV E1 and E2 proteins in membrane fusion and receptor interactions are limited, strong inferences can be drawn from their close relationship to RuV and the conserved functions of this viral group [19].

The E1 protein is essential for membrane fusion, functioning as a class II viral fusion protein similar to RuV. In the rubella virus, E1 mediates calcium-dependent fusion via low pH–induced conformational changes, and RusV E1 likely shares these conserved functional domains. The E2 protein facilitates receptor binding and viral attachment, forming a heterodimer with E1 that shields the fusion loops until fusion is triggered. Although RusV-specific data are lacking, its close phylogenetic relationship to RuV suggests that E1 and E2 retain analogous roles in host cell entry [33].

The biological barriers pertinent to zoonotic transmission, such as receptor compatibility and host immune responses, have not been fully elucidated for RusV. Like other animal viruses, RusV encodes receptor-binding proteins (RBPs) that mediate viral attachment and entry into host cells. Recent research highlights that RBPs from many animal viruses, including RusV, are broadly compatible with human cell entry factors. This suggests that RusV’s RBPs might potentially interact with human cellular receptors, facilitating viral entry, although direct evidence for RusV binding to specific human receptors is still lacking [34].

These findings emphasize the necessity of comprehensive screening of animal encephalitis cases and further research into the zoonotic potential of RusV. Considering its pathogenic potential and cross-species transmission capability, RusV should be recognized as a virus of emerging public health relevance.

### Comparison of RusV’s Zoonotic Risk with Other Emerging Neurotropic Viruses

Emerging neurotropic viruses represent significant public health concerns because of their capacity for zoonotic transmission, neuroinvasion, and high mortality rates. Although RusV’s zoonotic potential remains hypothetical, evaluating it in the context of established neurotropic viruses such as Borna disease virus 1 (BoDV-1) and Nipah virus (NiV) provides valuable context for assessing its possible threat to humans.

In contrast, BoDV-1 represents a well-established zoonotic pathogen with a clearer link to human disease than RusV. In veterinary medicine, BoDV-1–induced encephalitis is known as Borna disease (BD), primarily affecting horses and sheep. In humans, BoDV-1 is definitively associated with rare but often fatal cases of encephalitis. The natural reservoir of BoDV-1 is the bicolored white-toothed shrew (*Crocidura leucodon*), which harbors the virus without apparent disease, facilitating persistent virus circulation in the environment. Human infections are believed to arise from spillover events directly from this animal reservoir, although the precise modes and risk factors for transmission to humans remain incompletely understood. Moreover, while human-to-human transmission of BoDV-1 is generally considered rare, it has been recorded in organ transplant recipients, indicating that the virus can be transmitted between humans under specific circumstances [35].

NiV poses a particularly high zoonotic threat, causing mild respiratory illness as well as severe and often fatal encephalitis in humans. It is responsible for frequent and severe outbreaks, primarily in Southeast Asia, with case fatality rates ranging from 40% to 75%, highlighting its substantial lethality [36]. NiV is maintained in asymptomatic fruit bats (*Pteropus* spp.), facilitating persistent viral circulation and spillover. Human infection occurs via direct contact with infected animals or their secretions, consumption of contaminated food, or sustained human-to-human transmission, including in healthcare settings [37]. This combination of efficient human transmission and aggressive neurological and respiratory disease establishes NiV as a major public health hazard in endemic regions.

In summary, RusV currently represents a limited and uncertain zoonotic threat compared to BoDV-1 and NiV. Nonetheless, vigilant surveillance and further research are essential to clarify RusV’s zoonotic potential and contextualize its public health implications relative to these other neurotropic viruses.

## 7. Future Directions for the Field

Research on RusV is still in its early stages. While significant progress has been made in identifying it as the causative agent of staggering disease in cats and other mammals, many fundamental questions remain unanswered. Future studies are crucial to enhance understanding of this emerging pathogen and evaluate its potential implications for both animal and human health.

Currently, knowledge about viral shedding, transmission modes, and environmental persistence of RusV remains limited and warrants further investigation. One of the most pressing issues is identifying the virus’s natural reservoir and transmission routes. At present, wild rodents—particularly the yellow-necked field mouse in Germany and Austria, and the wood mouse in Sweden—are considered the most likely natural reservoirs of RusV [2,12]. Although most documented RusV infections have occurred in domestic cats, it remains unclear whether RusV primarily circulates in cats or whether they are incidental hosts infected through spillover from wildlife, such as rodents [2,3,4]. Furthermore, there is no direct evidence or documented case of vertical transmission (transplacental or perinatal) or vector-borne spread. Experimental infections—for example, infecting pregnant reservoir animals such as yellow-necked field mice, or experimentally exposing potential arthropod vectors (e.g., ticks, mosquitoes, fleas) to infected animals—could help clarify RusV transmission routes. However, this is currently not feasible, as no RusV isolates are available; thus, most data remain limited to in silico predictions and analogies with rubella virus (RuV). Moreover, the full host range and geographic distribution of RusV remain poorly defined, with most cases reported from restricted regions in Northern and Central Europe and, more recently, the United States [2,3,4,25]. This suggests that the virus may be more widespread than currently recognized.

Although RusV has been linked to nonsuppurative meningoencephalomyelitis in cats and other mammals, it is evident that not all cases of this disease can be attributed to RusV infection, indicating that other unidentified pathogens may be involved [38]. Effective diagnostic tools are urgently needed to identify RusV infections in both clinical and research settings. Molecular assays, serological tests, and immunohistochemical markers would support accurate case identification and epidemiological studies. To achieve this, further studies are required to isolate the virus and establish a suitable infection model.

## 8. Conclusions

In conclusion, the discovery of RusV highlights the urgent need to address emerging zoonotic threats. Proper monitoring and surveillance of zoonotic diseases are essential for gaining insights into the potential risks RusV may present to public health. RusV’s capacity to infect a broad range of mammalian hosts and its close genetic relationship to the rubella virus suggest a possible risk for cross-species transmission. The virus’s neurotropism and its presence in both domestic and wild animals underline the importance of continued surveillance and research. It is crucial to conduct comprehensive epidemiological studies, improve diagnostic tools, and adopt a One Health approach to fully understand the zoonotic potential of RusV and develop effective prevention strategies. Considering its biological characteristics and cross-species transmission capability, RusV should be regarded as an emerging pathogen that could pose a concern for both veterinary and public health.

## Figures and Tables

**Table 2:** The lowest identity/similarity in nucleotide and amino acid sequences of particular coding regions of the RusV isolates’ genomes.

	Comparable Fragment of the Genome	Nucleotide Sequence		Amino Acid Sequence		
		Identity	Gaps	Identity	Similarity	Gaps
All isolates *	**C**	760/1033 (73.6%)	71/1033 (6.9%)	273/333 (82.0%)	288/333 (86.5%)	1/333 (0.3%)
	**E1**	1100/1497 (73.5%)	57/1497 (3.8%)	393/487 (80.7%)	434/487 (89.1%)	0/487 (0.0%)
	**E2**	698/1038 (67.2%)	135/1038 (13.0%)	220/324 (67.9%)	256/324 (79.0%)	1/324 (0.3%)
	**p90**	1930/2535 (76.1%)	84/2535 (3.3%)	729/842 (86.6%)	768/842 (91.2%)	24/842 (2.9%)
	**p150**	2373/3464 (68.5%)	463/3464 (13.4%)	765/1107 (69.1%)	835/1107 (75.4%)	59/1107 (5.3%)
Clad 1 *	**C**	921/996 (92.5%)	0/996 (0.0%)	328/332 (98.8%)	328/332 (98.8%)	0/332 (0.0%)
	**E1**	1359/1464 (92.8%)	0/1464 (0.0%)	479/487 (98.4%)	481/487 (98.8%)	0/487 (0.0%)
	**E2**	888/972 (91.4%)	0/972 (0.0%)	316/324 (97.5%)	319/324 (98.5%)	0/324 (0.0%)
	**p90**	2295/2484 (92.4%)	0/2484 (0.0%)	812/828 (98.1%)	817/828 (98.7%)	0/828 (0.0%)
	**p150**	2999/3286 (91.3%)	14/3286 (0.4%)	1021/1093 (93.4%)	1030/1093 (94.2%)	0/1093 (0.0%)
Clad 2 *	**C**	824/1001 (82.3%)	7/1001 (0.7%)	314/333 (94.3%)	318/333 (95.5%)	1/333 (0.3%)
	**E1**	1212/1474 (82.2%)	11/1474 (0.7%)	464/490 (94.7%)	474/490 (96.7%)	3/490 (0.6%)
	**E2**	790/1040 (76.0%)	76/1040 (7.3%)	289/324 (89.2%)	307/324 (94.8%)	0/324 (0.0%)
	**p90**	2116/2489 (85.0%)	10/2489 (0.4%)	791/828 (95.5%)	806/828 (97.3%)	0/828 (0.0%)
	**p150**	2642/3320 (79.6%)	148/3320 (4.5%)	922/1086 (84.9%)	952/1086 (87.7%)	8/1086 (0.7%)

* The analysis was conducted on 35 strains listed in Supplementary Table S1, as well as on strains from clades 1 and 2 identified in the phylogenetic analysis below. The identity/similarity was calculated using the EMBOSS Needle Pairwise Sequence Alignment tool (https://www.ebi.ac.uk/jdispatcher/psa/emboss_needle) (accessed on 1 July 2025).

## Data Availability

All data are available in the manuscript.

## Supplementary Materials

The following supporting information can be downloaded at: https://www.mdpi.com/article/10.3390/pathogens14090851/s1, Table S1: RusV strains (*n* = 35) with complete genome sequences available in GenBank and used in the in silico analysis (accessed on 1 July 2025); Table S2: Estimates of evolutionary divergence between nucleotide sequences of 35 different RusV isolates obtained from the NCBI GenBank database. The number of base substitutions per site between sequences is shown. Analyses were conducted using the Maximum Composite Likelihood model [39]. The analysis involved 35 nucleotide sequences. All ambiguous positions were removed for each sequence pair (pairwise deletion option). There were a total of 9768 (whole genome), 984 (C protein), 1462 (E1), 965 (E2), 2501 (p90), and 3341 (p150) positions in the final dataset. Evolutionary analyses were conducted in MEGA11 [31]; Table S3: Estimates of evolutionary divergence between amino acid sequences of 35 different RusV isolates obtained from the NCBI GenBank database. The number of amino acid substitutions per site between sequences is shown. Analyses were conducted using the Poisson correction model [40]. The analysis involved 35 amino acid sequences. All ambiguous positions were removed for each sequence pair (pairwise deletion option). There were a total of 301 (C protein), 487 (E1), 297 (E2), 759 (p90), and 1041 (p150) positions in the final dataset. Evolutionary analyses were conducted in MEGA11 [31].

## Abbreviations

The following abbreviations are used in this manuscript:

RusV	Rustrela virus
SD	Staggering disease
RuV	Rubella virus
RuhV	Ruhugu virus
BoDV	Borna disease virus
FFPE	Formalin-fixed, paraffin-embedded
ICTV	International Committee on Taxonomy of Viruses
ORF	Open reading frame
nsPP	Non-structural polyprotein
sPP	Structural polyprotein
RdRP	RNA-dependent RNA polymerase
E1	Envelope glycoprotein 1
E2	Envelope glycoprotein 2
C	Capsid protein
HTS	High-throughput sequencing
VNT	Virus neutralization test
