# Supplementary material for: Newly Discovered Rustrela Virus: Current State of Knowledge About the Etiological Agent of Feline “Staggering Disease”
Source: Pathogens. 2025 Aug 27;14(9):851. doi: 10.3390/pathogens14090851 (PMC12472204; doi:10.3390/pathogens14090851)
Supplement: Supplementary file 1 [file pathogens-14-00851-s001.zip › Supplementary Table S1.pdf]

Supplementary Table S1. RusV strains (n = 35) with complete genome sequences available in GenBank and used in the *in silico* analysis (accessed July 2025).

Strain designation	Gen Bank No.	Host	Source	Country of isolation	Year of isolation
Yellow-necked field mouse/Mu09-1341/2009/Germany	OL960721.1	*Apodemus flavicollis*	Brain	Germany	2009
RusVs/yellow-necked field mouse/MV.DEU/KS20-2266/2013	OP689523.1	*Apodemus flavicollis*	Brain	Germany	2013
RusVs/yellow-necked field mouse/MV.DEU/KS20-2226/2015	OP689524.1	*Apodemus flavicollis*	Brain	Germany	2015
RusVs/yellow-necked field mouse/MV.DEU/KS20-2242/2015	OP689517.1	*Apodemus flavicollis*	Brain	Germany	2015
RusVs/yellow-necked field mouse/MV.DEU/KS20-1592/2016	OP689526.1	*Apodemus flavicollis*	Brain	Germany	2016
RusVs/yellow-necked field mouse/MV.DEU/KS20-1610/2016	OP689525.1	*Apodemus flavicollis*	Brain	Germany	2016
RusVs/yellow-necked field mouse/MV.DEU/KS20-2273/2016	OP689522.1	*Apodemus flavicollis*	Brain	Germany	2016
RusVs/yellow-necked field mouse/MV.DEU/KS20-1655/2016	OP689519.1	*Apodemus flavicollis*	Brain	Germany	2016
RusVs/yellow-necked field mouse/MV.DEU/KS20-2346/2016	OP689521.1	*Apodemus flavicollis*	Brain	Germany	2016
RusVs/yellow-necked field mouse/MV.DEU/KS20-2189/2016	OP689518.1	*Apodemus flavicollis*	Brain	Germany	2016
Yellow-necked field mouse/KS19-928/2019/Germany	MT274725.2	*Apodemus flavicollis*	Brain	Germany	2019
RusVs/yellow-necked field mouse/BB.DEU/KS20-1455/2019	OP689520.1	*Apodemus flavicollis*	Brain	Germany	2019
Yellow-necked field mouse/KS20-1296/2019/Germany	OL960722.1	*Apodemus flavicollis*	Brain	Germany	2019
Yellow-necked field mouse/KS20-1340/2020/Germany	OL960726.1	*Apodemus flavicollis*	Brain	Germany	2020
Yellow-necked field mouse/KS20-1535/2020/Germany	OL960718.1	*Apodemus flavicollis*	Brain	Germany	2020

Yellow-necked field mouse/KS20-1342/2020/Germany	OL960724.1	*Apodemus flavicollis*	Brain	Germany	2020
Yellow-necked field mouse/KS20-1513/2020/Germany	OL960719.1	*Apodemus flavicollis*	Brain	Germany	2020
Yellow-necked field mouse/KS20-1341/2020/Germany	OL960725.1	*Apodemus flavicollis*	Brain	Germany	2020
Yellow-necked field mouse/KS20-1512/2020/Germany	OL960720.1	*Apodemus flavicollis*	Brain	Germany	2020
Yellow-necked field mouse/KS20-1343/2020/Germany	OL960723.1	*Apodemus flavicollis*	Brain	Germany	2020
RusVs/wood mouse/SWE/KS21-1349/1996	ON641047.1	*Apodemus sylvaticus*	Brain	Sweden	1996
RusVs/wood mouse/SWE/KS21-1358/2005	ON641048.1	*Apodemus sylvaticus*	Brain	Sweden	2005
RusVs/wood mouse/SWE/KS21-1362/2011	ON641049.1	*Apodemus sylvaticus*	Brain	Sweden	2011
RusVs/red-necked wallaby/BB.DEU/21_105/2021	OP221677.1	*Notamacropus rufogriseus*	Brain	Germany	2021
RusVs/red-necked wallaby/MV.DEU/21_136/2021	OP221675.1	*Notamacropus rufogriseus*	Brain	Germany	2021
South American Coati/20_131/2020/Germany	OL960717.1	*Nasua nasua*	Brain	Germany	2020
Eurasian otter/21_002/2020/Germany	OL960716.1	*Lutra lutra*	Brain	Germany	2020
Donkey/19_041-1/2019/Germany	NC_076451.1	*Equus asinus*	Brain	Germany	2019
Capybara/P19-643/2019/Germany	MT274724.2	*Hydrochoerus hydrochaeris*	Brain	Germany	2019
RusVs/Cougar/CO.USA/WHL23887/2023	PP025855.1	*Puma concolor*	Brain	USA	2023
RusVs/cat/AUT/AUT_02/1992	ON641041.1	*Felis catus*	Brain	Austria	1992
RusVs/cat/MV.DEU/GER_04/2021	ON641043.1	*Felis catus*	Brain	Germany	2021
RusVs/cat/SWE/SWE_13/2021	ON641044.1	*Felis catus*	Brain	Sweden	2021
RusVs/cat/SWE/SWE_14/2021	ON641045.1	*Felis catus*	Brain	Sweden	2021
RusVs/cat/SWE/SWE_15/2021	ON641046.1	*Felis catus*	Brain	Sweden	2021
